# Inequalities in mental health service utilisation by children and young people: a population survey using linked electronic health records from Northwest London, UK

**DOI:** 10.1136/jech-2023-221223

**Published:** 2023-12-12

**Authors:** Antonio Ivan Lazzarino, Jessica Ann Salkind, Federica Amati, Tamsin Robinson, Shamini Gnani, Dasha Nicholls, Dougal Hargreaves

**Affiliations:** 1 Department of Epidemiology and Biostatistics, Imperial College London, London, UK; 2 The Whittington Hospital, London, UK; 3 NIHR ARC NWL Children and Young People’s Mental Health Network, Imperial College London, London, UK; 4 Department of Primary Care and Public Health, Imperial College London, London, UK; 5 NHS Northwest London Integrated Care Board, London, UK; 6 Department of Brain Sciences, Imperial College London, London, UK

**Keywords:** MENTAL HEALTH, HEALTHCARE DISPARITIES, HEALTH SERVICES, PUBLIC HEALTH

## Abstract

**Background:**

Mental healthcare services for children and young people (CYP) are a very limited resource in the UK. To prevent health inequalities, measures to increase overall capacity must sit alongside measures that ensure utilisation matches need.

**Aim:**

Our aim was to identify subgroups of CYP with unexpectedly low mental health service utilisation, presumably representing unmet need, and to assess whether there is area variation in the socioeconomic gradient of mental healthcare use.

**Methods:**

This is a cross-sectional population survey of CYP (aged 5–24 years) using electronic health records from the Discover Now research platform, covering approximately 95% of the Northwest London resident population of 2.4 million people.

**Results:**

The total sample comprised 764 327 CYP, of whom 2.1% attended a mental healthcare appointment in 2021 (95% CI 2.1% to 2.2%), our outcome measure. Lower socioeconomic status (our main exposure factor) was related to higher occurrence of mental healthcare appointments (+5% for each quintile increase in deprivation (95% CI 2% to 7%, p<0.001]). However, interaction analyses showed that the boroughs with unexpectedly low utilisation rates were also those not showing a clear trend between socioeconomic conditions and services utilisation (interaction p<0.001), suggesting that in these boroughs the occurrence of mental disorders in disadvantaged people was not captured by our analysis based on service utilisation. In some London boroughs, we found lower-than-expected activity for the most disadvantaged CYP.

**Conclusions:**

The mental healthcare needs of many CYP from socioeconomically deprived areas of Northwest London may be unmet. More information is needed to confirm our results.

**WHAT IS ALREADY KNOWN ON THIS TOPIC**

- The risk of mental health conditions is higher in socioeconomically deprived people. In the UK, only around 30% of children and young people (CYP) with a mental health condition can benefit from treatment and support.

**WHAT THIS STUDY ADDS**

- In some London boroughs, the activity of specialist mental health centres is higher for CYP from disadvantaged backgrounds, while in other boroughs this socioeconomic gradient is flat, suggesting an unmet need.

**HOW THIS STUDY MIGHT AFFECT RESEARCH, PRACTICE OR POLICY**

- The present study addresses a key priority for the Northwest London Integrated Care Board and can directly inform local policies. In addition, this study contributes to the implementation of the 2019 National Health Service (NHS) long-term plan, which includes the expansion of timely and age-appropriate mental health services, and the identification of selected areas where additional resources/facilities are to be provided.

## Background

Childhood and adolescence are critical times for the emergence of mental health conditions which closely interlink with physical health, educational attainment and future life outcomes.^1^ Fifty per cent of mental health problems are established by the age of 14% and 75% by the age of 24.^2^ Timely access to appropriate mental health support is vital as early intervention has been shown to improve long-term outcomes and delaying access may mean longer or more intensive treatments are needed.^3^ In recent years in England, there have been well-documented rising rates of mental health problems in children and young people (CYP), with long waiting lists and rejection of referrals made to CYP’s mental health services.^4^

This situation appears to have worsened since the COVID-19 pandemic with nationally representative surveys conducted by NHS Digital showing significant increases in rates of self- and parent-reported mental disorder compared with prepandemic data.^5^ In children aged 7–16 years, they found rates of ‘probable mental disorder’ rose from 12.1% in 2017 to 16.7% in 2020. In the older group aged 17–19 years, rates rose from 10.1% in 2017 to 17.7% in 2020, followed by another rise to 25.7% in 2022. This trend can be understood in terms of the multifaceted impact of the COVID-19 pandemic on mental health and well-being, with extend periods of lockdown, school closures and social distancing, preventing CYP accessing education and social activities, alongside financial implications for families.^6^

The increase in the need for mental health support due to the pandemic has not been mirrored by a sufficient equivalent rise in availability of mental health services for CYP. The 2022 Office of the Children’s Commissioner annual report on mental health services for CYP across England found markers of improvement at a national level with a real terms increase in spending on mental health services for CYP by 4.4% from 2019/2020 to 2020/2021, and a reduction in average waiting times from 53 days in 2018/2019 to 32 days in 2020/2021.^4^ Despite this, the authors estimated the access rate of CYP with a probable mental disorder to mental health services for CYP to be just 32%. In addition, for the first time in recent years, there has been a decrease in referral rates from 539 000 (4.5%) of under 18s in 2019/2020 to 497 502 (4%) in 2020/2021. The reduction in referrals may reflect reduced access of CYP to education and primary care during the pandemic, key settings for referral to mental health services for CYP.

With an overall picture at a national level of a mental health service unable to meet the needs of its CYP, there is also evidence of stark geographical inequality in mental health services for CYP spending and access across the country. The Children’s Commissioner 2022 report found only 50% of Clinical Commissioning Groups (CCGs) had reached the NHS Long Term Plan benchmark of spending at least 1% of overall CCG budget on mental health services for CYP.^4^ Comparing different CCGs, Children and Young People's Mental Health Service (CYPMHS) spending per child varied from £16 to 165 and average wait time to enter treatment varied from 6 to 81 days.

Utilisation of mental healthcare services in a particular area is influenced by a complex interaction between availability of services, barriers to accessing care (also including stigma, health literacy, information barriers and cultural issues) and the socioeconomic characteristics of the local population. These characteristics can in turn impact on prevalence of mental disorder, healthcare-seeking behaviour and likelihood of referral. Overall, areas with unexpectedly low utilisation of mental health services are presumably areas with significant unmet need.

As mental health services for CYP are a very limited resource, measures to increase overall capacity must sit alongside measures to best target this limited resource to those with the highest need and ensure health inequalities are reduced.

In this study, we describe how rates of access of CYP to mental health services vary across different geographical regions (eight boroughs of Northwest London) and how these rates differ with socioeconomic deprivation. In doing so, we aim to identify specific subgroups within boroughs with unexpectedly low mental health service utilisation, presumably representing unmet need, and to assess whether there is area variation in the socioeconomic gradient of mental healthcare use.

## Methods

### Study design

Cross-sectional population survey using linked electronic health records.

### Setting

The Northwest London Integrated Care System has attempted to collect all health-related information from all people who are resident in that area of the UK within a database called Whole Systems Integrated Care (WSIC), with the main objective of improving the quality of care by making individual-level health information readily available to health professionals when visiting patients.^7^

This study is based on the analysis of deidentified WSIC records, which are made available to researchers on request through a research platform called Discover Now.^8^ Discover Now therefore gathers individual-level electronic health records from primary, secondary and tertiary care for all people registered with a family doctor or a general practitioner (GP) in North West London, covering approximately 95% of the resident population of 2.4 million people. Discover Now has been in operation since 2015 and gathers information from 365 GP practices, 10 acute and specialist hospitals, 2 mental health trusts, 2 community health trusts and social care providers.^8^ Table 1 shows the data source tables that are available to researchers. Researchers can link the source tables together using deidentified NHS numbers. Data source refreshes are scheduled every 1–3 months depending on the data source. For the present analysis, the data were extracted in January 2023 (table 1).

**Table 1:** Individual-level, deidentified, data source tables from the discover now platform available to researchers, at April 2023

Original data source tables	Derived data source tables
Community	Asthma Activity
GPNetworks_Earlyadopters	Asthma Radar
High Cost Drugs	COPD Patient Radar
Mental Health	COPD Unconfirmed Patient Radar
Patient Index	Diabetes Benchmarking
Prescriptions	Diabetes Risk
Primary Care	Learning Disability Dashboard
SLAM	Long Term Conditions
Social Care	
SUS A&E	
SUS Episodes	
SUS Out Patients	
SUS Spells	

### Participants

Northwest London residents, who were aged 5–24 years in the year 2021.

### Eligibility criteria

All CYP registered at a general practice in Northwest London were included. We excluded CYP registered with the ‘GP at Hand’ practice in Hammersmith & Fulham as it has registered patients outside Northwest London (providing digital consultations via a phone app).

### Outcome measure

Our primary outcome measure was attendance at one or more planned outpatient appointments at one of the two mental healthcare provider trusts in the year 2021 (hereon referred to as ‘MH appointment’). In the UK, patients mainly access specialist (secondary) care, including mental healthcare, through their primary care provider (GP referral or school/college).

The trusts provide specialist child and adolescent mental health services as well as other targeted services, for example, eating disorders services and some mental health in schools’ teams. The Discover Now research platform does not capture, however, the mental healthcare activity from outside the mental health provider trusts’ remit, such as counselling (including school counselling) services or other community mental health support.

### Variable derivation and linkage

We used the table *Patient Index* to identify a cohort of people who were aged 5–24 years in the year 2021. We used the table *GPNetworks_Earlyadopters* to derive information about the GP practices and Primary Care Networks at which individuals were registered. We then used the table *Mental Health* to identify which people attended an outpatient appointment at either of the two mental health provider trusts located in Northwest London in 2021. We classified the activity records from mental health trusts as being outpatient appointments when the variable *Dataset* took the value of either *Community*, *Outpatient* or *Day Case*. Appointment dates were derived using the variable *Contact Date*.

### Exposure variables

The WSIC table *Patient Index* includes sociodemographic information including gender, borough of residence, ethnicity and socioeconomic deprivation. There are eight boroughs or districts in Northwest London: Ealing, Brent, Hillingdon, Hounslow, Harrow, Hammersmith & Fulham, Westminster, and Kensington & Chelsea. Ethnicity takes five possible values (variable named *EthnicCategory*): Asian, black, mixed, other and white. Ethnicity was unspecified for 5.8% of the study sample, and we included those people in the analysis, considering them as a sixth category (unspecified ethnicity).

For socioeconomic deprivation, the variable named *imdrank* indicates the English Index of Multiple Deprivation (IMD) for year 2019, which is an area-based index of poverty produced by the UK Ministry of Housing, Communities and Local Government, which is calculated as a weighted score based on seven domains: income, employment, education, health, crime, barriers to housing and services, and living environment.^9^ A higher score indicates lower socioeconomic deprivation. In this study, the score was divided into quintiles, IMD1–5 with IMD 1 representing the most socioeconomically deprived group.

### Statistical analysis

We calculated crude mental health (MH) appointment attendance rates with 95% CIs for the whole sample and after stratification by sociodemographic characteristics including age, gender, ethnicity, borough of residence and IMD quintile. For borough and ethnicity, we used the most populated category as the reference category. This happened to be Ealing for the borough of residence, and white for the ethnicity. We focused our analysis on the interaction between borough of residence and IMD quintile to evaluate whether the socioeconomic gradient of mental healthcare use differs between borrows. Interaction analyses and stratum-specific MH appointment rates were then adjusted using logistic regression and indirectly standardising to the observed distribution of covariates for the whole sample using the *margins* command in Stata V.15.

## Results

We identified 928 662 people who were aged 5–24 years in 2021. We excluded 159 517 people who were not resident in one of the eight boroughs of Northwest London and a further 4705 people who were registered with the GP practice called GP at Hand which has registered patients from outside Northwest London. We also excluded 113 people with missing values (n=90, gender) and (n=23, unspecified primary care network). The final analytic sample comprised 764 327 CYP aged between 5 and 24 years. The prevalence of attendance at a mental healthcare outpatient appointment in 2021 was 2.1% (95% CI 2.1% to 2.2%).

**Figure 1:**
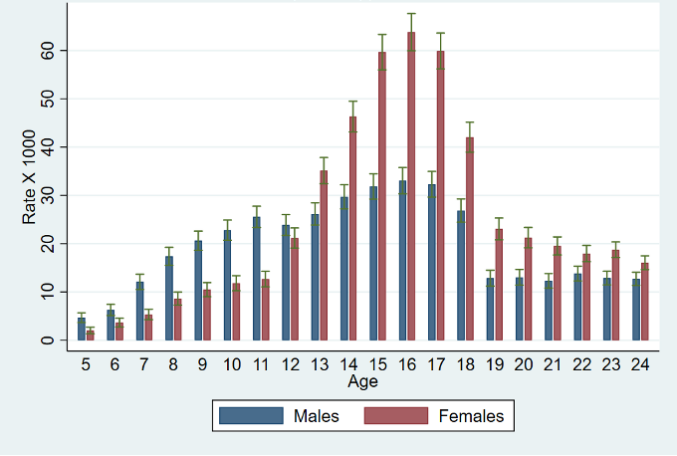
Utilisation rates of outpatient mental healthcare services in 2021 by age and gender, with 95% CIs.

Table 2 shows the variation in MH appointment rates by age, gender, ethnicity, socioeconomic deprivation and borough of residence. Age was associated with MH appointment rates, with older age being associated with higher rates, but the shape of the association appeared to be different between males and females and to change over time (figure 1).

Table 3 describes the covariates by borough of residence. All socioeconomic strata are represented in each Northwest London borough, although there was more deprivation in certain ones.

**Table 2:** Sample description of Discover Now/WSIC participants aged 5–24 years in 2021

Factor and category		Outpatient mental health service used in 2021		P value
		No	Yes	
		N=748 011	N=16 316	
Age in years		15.0 (6.0)	15.7 (4.6)	<0.001
Age category	5–12	291 169 (98.7%)	3951 (1.3%)	<0.001
	13–17	161 586 (95.9%)	6933 (4.1%)	
	18–24	295 256 (98.2%)	5432 (1.8%)	
Gender	Female	369 376 (97.6%)	8945 (2.4%)	<0.001
	Male	378 635 (98.1%)	7371 (1.9%)	
Ethnicity	White	280 006 (97.2%)	8019 (2.8%)	<0.001
	Asian	203 876 (98.7%)	2759 (1.3%)	
	Other	107 525 (98.2%)	1947 (1.8%)	
	Black	72 864 (97.4%)	1953 (2.6%)	
	Unspecified	44 136 (99.6%)	187 (0.4%)	
	Mixed	39 604 (96.5%)	1451 (3.5%)	
Socioecon. deprivation (quintile of IMD)	1 (most deprived)	147 438 (97.2%)	4311 (2.8%)	<0.001
	2	149 635 (97.8%)	3385 (2.2%)	
	3	155 541 (98.0%)	3173 (2.0%)	
	4	147 843 (98.1%)	2917 (1.9%)	
	5 (least deprived)	147 554 (98.3%)	2530 (1.7%)	
NWL Borough	Ealing	126 845 (97.7%)	2957 (2.3%)	<0.001
	Brent	127 224 (98.3%)	2253 (1.7%)	
	Hillingdon	117 909 (98.1%)	2276 (1.9%)	
	Hounslow	96 304 (97.1%)	2890 (2.9%)	
	Harrow	95 750 (97.9%)	2068 (2.1%)	
	Hammersmith & Fulham	66 125 (97.7%)	1554 (2.3%)	
	Westminster	65 474 (98.0%)	1310 (2.0%)	
	Kensington & Chelsea	52 380 (98.1%)	1008 (1.9%)	

Data are means (SD) for continuous variables and N (row percentage) for categorical variables. P values are from independent T-tests for continuous variables and from χ^2^ test for categorical variables.

NWL, NW London; WSIC, Whole Systems Integrated Care.

**Table 3:** Sample description of Discover Now/WSIC participants aged 5–24 years in 2021

Factor and category	Northwest London borough (local health authority)							
	Ealing	Brent	Hill.	Houns.	Harrow	H&F	Westm.	K&C
	N=129 802	N=129 477	N=120 185	N=99 194	N=97 818	N=67 679	N=66 784	N=53 388
Age in years*	14.8 (5.9)	15.0 (5.9)	14.7 (5.9)	14.3 (5.9)	14.6 (5.8)	15.9 (6.2)	16.1 (6.1)	15.8 (6.1)
Age category*								
5–12	50 974 (39.3%)	49 920 (38.6%)	48 515 (40.4%)	42 721 (43.1%)	40 021 (40.9%)	23 369 (34.5%)	21 472 (32.2%)	18 128 (34.0%)
13–17	29 428 (22.7%)	29 641 (22.9%)	27 672 (23.0%)	23 042 (23.2%)	23 476 (24.0%)	12 572 (18.6%)	12 232 (18.3%)	10 456 (19.6%)
18–24	49 400 (38.1%)	49 916 (38.6%)	43 998 (36.6%)	33 431 (33.7%)	34 321 (35.1%)	31 738 (46.9%)	33 080 (49.5%)	24 804 (46.5%)
Gender*								
Female	63 583 (49.0%)	64 076 (49.5%)	57 474 (47.8%)	47 890 (48.3%)	47 111 (48.2%)	35 976 (53.2%)	34 506 (51.7%)	27 705 (51.9%)
Male	66 219 (51.0%)	65 401 (50.5%)	62 711 (52.2%)	51 304 (51.7%)	50 707 (51.8%)	31 703 (46.8%)	32 278 (48.3%)	25 683 (48.1%)
Ethnicity*								
White	45 000 (34.7%)	39 292 (30.3%)	46 774 (38.9%)	33 327 (33.6%)	30 559 (31.2%)	37 567 (55.5%)	28 868 (43.2%)	26 638 (49.9%)
Asian	39 471 (30.4%)	37 674 (29.1%)	39 719 (33.0%)	32 892 (33.2%)	39 100 (40.0%)	4769 (7.0%)	8870 (13.3%)	4140 (7.8%)
Other	18 639 (14.4%)	19 502 (15.1%)	11 811 (9.8%)	9999 (10.1%)	11 641 (11.9%)	10 297 (15.2%)	15 993 (23.9%)	11 590 (21.7%)
Black	13 689 (10.5%)	20 252 (15.6%)	10 887 (9.1%)	7160 (7.2%)	7494 (7.7%)	7343 (10.8%)	4313 (6.5%)	3679 (6.9%)
Unspecified	6030 (4.6%)	6132 (4.7%)	4932 (4.1%)	11 375 (11.5%)	4370 (4.5%)	3198 (4.7%)	4476 (6.7%)	3810 (7.1%)
Mixed	6973 (5.4%)	6625 (5.1%)	6062 (5.0%)	4441 (4.5%)	4654 (4.8%)	4505 (6.7%)	4264 (6.4%)	3531 (6.6%)
Socioeconomic deprivation* (quintile of IMD)								
1 (most deprived)	31 777 (24.5%)	39 370 (30.4%)	16 498 (13.7%)	14 778 (14.9%)	4718 (4.8%)	18 716 (27.7%)	13 069 (19.6%)	12 823 (24.0%)
2	34 560 (26.6%)	30 242 (23.4%)	28 505 (23.7%)	29 659 (29.9%)	8690 (8.9%)	9642 (14.2%)	7141 (10.7%)	4581 (8.6%)
3	20 463 (15.8%)	36 544 (28.2%)	24 814 (20.6%)	22 697 (22.9%)	21 518 (22.0%)	13 448 (19.9%)	10 548 (15.8%)	8682 (16.3%)
4	26 045 (20.1%)	18 514 (14.3%)	17 961 (14.9%)	23 543 (23.7%)	32 553 (33.3%)	14 396 (21.3%)	9757 (14.6%)	7991 (15.0%)
5 (least deprived)	16 957 (13.1%)	4807 (3.7%)	32 407 (27.0%)	8517 (8.6%)	30 339 (31.0%)	11 477 (17.0%)	26 269 (39.3%)	19 311 (36.2%)

Data are means (SD) for continuous variables and N (column percentage) for categorical variables. P values are from Analysis of Variance (ANOVA) for continuous variables and from χ^2^ test for categorical variables.

*p<0.001.

H&F, Hammersmith & Fulham; Hill, Hillingdon; Houns, Hounslow; K&C, Kensington & Chelsea; West, Westminster.

**Figure 2:**
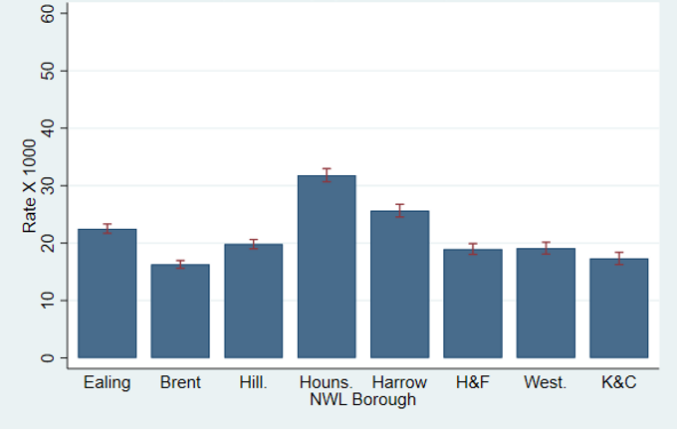
Utilisation rates of outpatient mental healthcare services in 2021, stratified by Northwest London (NWL) borough and adjusted for age, gender, socioeconomic deprivation and ethnicity, with 95% CIs. H&F, Hammersmith & Fulham; Houns., Hounslow; Hill., Hillingdon; IMD, Index of Multiple Deprivation; K&C, Kensington & Chelsea; West., Westminster.

Figure 2 shows MH appointment rates by borough after standardising for all covariates (age, gender, socioeconomic deprivation and ethnicity). The covariates could not explain most of the between-borough variation that was observed. Although boroughs from the outskirts of London such as Ealing, Harrow and Brent had roughly similar rates compared with central London boroughs such as Hammersmith & Fulham, Kensington & Chelsea, and Westminster, most confidence intervals in figure 2 do not overlap. So, for example, there is evidence that Ealing’s MH appointment rate was higher than Brent’s one, which was lower that Hillingdon’s one. Harrow’s rate was lower than Hounslow’s but higher than Hammersmith & Fulham’s, etc.

Table 4 shows the output from a multi-adjusted logistic regression model with interaction parameters between each borough and IMD quintile treated as a linear (ordered categorical) exposure, with score 1 meaning most deprived and score 5 meaning least deprived. There was strong evidence of a difference between the boroughs in terms of the effect that IMD had on their MH appointment rates, with p values <0.001. For example, CYP from Hounslow had twice the odds of attending an MH appointment compared with CYP from Ealing (OR 2.01, 95% CI 1.78 to 2.26, p<0.001), with a steeper slope quantifying the association between IMD and odds of MH appointment (OR for each IMD quintile increase 0.88, 95% CI 0.84 to 0.91, p<0.001). Since the association between age and MH appointment rates was not linear and differed by gender, we carried out a sensitivity analysis by fitting the same model again after including an interaction parameter between gender and age treated as a categorical variable. The key coefficients were not substantially changed (output in online supplemental appendix 1).

10.1136/jech-2023-221223.supp1 Supplementary data

**Table 4:** Output from a multiple logistic regression model showing mutually adjusted ORs for the utilisation of outpatient mental healthcare services in 2021 (OR), with 95% CIs and p values

Factor	Category	OR (95% CI)	P value
Borough	Ealing	1 (Reference category)	
	Brent	0.82(0.73 to 0.93)	0.001
	Hillingdon	0.90(0.80 to 1.03)	0.12
	Hounslow	2.01(1.78 to 2.26)	<0.001
	Harrow	1.15(0.97 to 1.35)	0.10
	Hammersmith & Fulham	1.25(1.10 to 1.43)	0.001
	Westminster	1.71(1.50 to 1.96)	<0.001
	Kensington & Chelsea	1.57(1.36 to 1.81)	<0.001
IMD (1=most deprived; 5=least deprived)	1 quintile increase	0.95(0.93 to 0.98)	0.001
Interaction Borough×IMD	Ealing	1 (Reference category)	
(Slope of IMD for each Borough)	Brent	0.96(0.92 to 1.00)	0.068
	Hillingdon	0.98(0.94 to 1.02)	0.24
	Hounslow	0.88(0.84 to 0.91)	<0.001
	Harrow	0.97(0.93 to 1.02)	0.23
	Hammersmith & Fulham	0.86(0.82 to 0.90)	<0.001
	Westminster	0.78(0.74 to 0.81)	<0.001
	Kensington & Chelsea	0.76(0.73 to 0.80)	<0.001
Gender	Male	0.82(0.79 to 0.84)	<0.001
Ethnicity	White	1 (Reference category)	
	Asian	0.44(0.42 to 0.46)	<0.001
	Other	0.62(0.59 to 0.65)	<0.001
	Black	0.85(0.80 to 0.89)	<0.001
	Unspecified	0.13(0.12 to 0.15)	<0.001
	Mixed	1.26(1.19 to 1.33)	<0.001
Age	1 year increase	1.03(1.02 to 1.03)	<0.001

**Figure 3:**
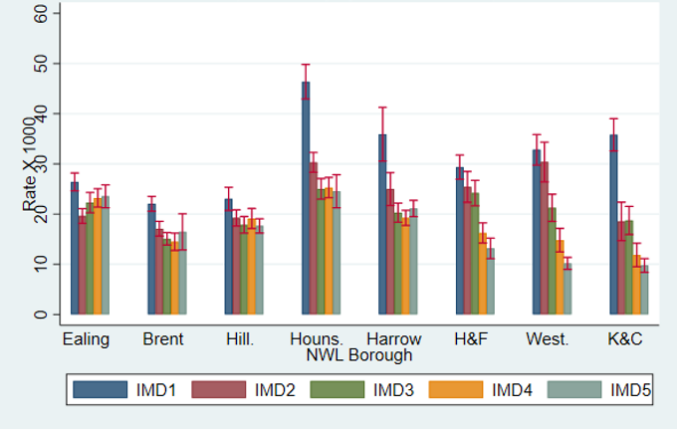
Utilisation rates of outpatient mental healthcare services in 2021, stratified by Northwest London (NWL) borough and socioeconomic deprivation quintile, adjusted for age, gender and ethnicity, with 95% CIs. Note: IMD1=most deprived; IMD5=least deprived. H&F, Hammersmith & Fulham; Houns., Hounslow; Hill., Hillingdon; IMD, Index of Multiple Deprivation; K&C, Kensington & Chelsea; West., Westminster.

Figure 3 shows MH appointments rates by borough of residence and quintile of socioeconomic deprivation, after adjusting for age, gender and ethnicity. The association between socioeconomic deprivation and MH appointment rates differed by borough. In central London boroughs such as Hammersmith & Fulham, Westminster, and Kensington & Chelsea, the trend of lower rates of appointments in the least deprived groups and the higher rates of the most deprived ones was more pronounced. For the boroughs of Ealing, Brent and Harrow, there was no clear evidence of that trend (figure 3).

## Discussion

In this study, we compared the use of mental healthcare services among the eight boroughs of Northwest London, measured as attended outpatient appointments for CYP aged 5–24 years, with the aim of assessing whether there is a locality variation in the socioeconomic gradient of mental healthcare use. To our knowledge, this is the first time such an approach has been taken to examining socioeconomic disparities in access to mental healthcare, and equivalent analyses of those reported in this study have not been carried out elsewhere.

This study follows the NHS long-term plan 2019.^10^ Under that plan, the NHS made a new commitment to improve mental health services for CYP. It was estimated that in the UK only around 30% of CYP with a mental health condition can benefit from treatment and support. There is a need for expanding timely, age-appropriate services to reduce pressures on emergency departments, paediatric wards and ambulance services. The NHS also committed to identify selected areas where extra resources/structures are to be provided.^10^ This study can inform the NHS on the actions to be taken to fulfil the mission stated in the NHS long term plan.

We expected higher utilisation rates in the suburbs more distant from central London, such as Ealing, Brent, Hillingdon, Hounslow and Harrow, compared with central London’s more affluent boroughs such as Hammersmith & Fulham, Westminster, and Kensington & Chelsea. While this was the case for Hounslow and Harrow, the other less affluent suburbs had utilisation rates similar to those in central London, both before and after adjustments (figure 3). In Brent, for example, only 3.5% of people lived in an area with an index of deprivation of 5, indicating least deprivation. This is 10 times less compared with central London areas such as Kensington, for example (table 3). Despite of that, the secondary mental healthcare activity levels of those two boroughs were similar (table 2).

Socioeconomic deprivation impacts CYP’s mental health through adverse childhood experiences, poverty, discrimination and a lack of support, and is therefore associated higher prevalence of mental disorders.^11^ However, judging from mental healthcare use, in our sample this trend was evident only for some boroughs, and high utilisation rate was not always associated with areas of lower deprivation. The stratified analysis showed that the boroughs with unexpectedly low utilisation rates also did not show a clear association between socioeconomic deprivation and mental health services utilisation, even when adjusted for other factors such as age, gender and ethnicity. The causes of these disparities are likely to be multifaceted and merit consideration within the Northwest London population health survey. For example, there may be some displacement effect of non-NHS services for more affluent groups, differences in investment by borough in mental health provision, differences in willingness to access mental healthcare in some populations or differences in community and social care support that influence the need for mental health treatment.

Nonetheless, our findings are consistent with high levels of unmet need among CYP living in the deprived areas of this part of London (Ealing, Brent and Hillingdon) when compared with the deprived areas from central London. The higher rates of MH appointments in Hounslow and Harrow are accounted for by the very high rates of their most deprived areas, which were not manifest in Ealing, Brent and Hillingdon, suggesting that in these boroughs the occurrence of mental disorders in the most disadvantaged people was not captured by our analysis based on service utilisation.

In other words, what is surprising in figure 3 is the absence of high utilisation rates in the most deprived areas of Ealing, Brent and Hillingdon. The mental healthcare activity dedicated to the most disadvantaged CYP seem to be missing in those boroughs. These findings are compatible with differentiated policies between boroughs or primary care networks, as it is known that some London boroughs have higher staffing while other ones show longer waiting lists.^12^

### Limitations

The main limitation to the present analysis is that we did not have information on the activity from mental health support teams in schools nor from a number of community or early intervention MH services commissioned by Local Authorities. The Discover Now research platform does not capture that information today. Our results could therefore be explained by higher mental health support from schools, social care, or other MH services commissioned by the Local Authority of some boroughs.

Deprivation was measured using an area-based index, IMD, which was calculated by a third party, institutional body (Ministry of Housing). Our results could be biased if IMD scores from outer London were more distorted compared with central London, if, for example, they were influenced by factors linked to social diversity. For instance, employment is a major component of the IMD index, weighting 22.5% of the score. If being unemployed does not produce a psychosocial disadvantage in certain environments or cultures, the poverty of those areas may be overestimated.

Furthermore, we did not have data from private services or services used abroad, which may contribute to the lower reported utilisation rates of NHS services in some affluent areas. This cannot however explain the lower-then-expected activity from the deprived areas of certain boroughs.

Mental health trusts adopt policies defining thresholds for access to services. Our results could possibly be explained by the fact that the referrals for certain groups did not meet the service access thresholds.

There are two mental health provider trusts in Northwest London. Any differences in policies between the trusts cannot explain the different utilisation rates between boroughs, as the discrepancies are evident even for boroughs sharing the same provider trust, such as Hounslow and Ealing for example.

We have no data for CYP who were referred to mental health services and were not brought to/did not attend appointments. CYP from the less affluent areas of certain boroughs may have more barriers to mental healthcare access. Moreover, our analysis concerned outpatient appointments, and therefore did not include inpatient psychiatric care nor the activity from mental health crisis teams in acute settings. One could argue that CYP with more barriers to accessing planned mental healthcare are more likely to experience a deterioration of their condition and are more likely to experience severe acute episodes. These extra episodes would only partially explain the lack of planned MH appointments that we found in the most deprived sectors of certain boroughs. Moreover, if sectors of the population have barriers to the use of a health service, this is still a form of inequality, regardless of whether the referral was made or not. Therefore, the fact that our data did not include CYP who did not use mental health services despite having an appointment booked, as well as those who used acute crisis teams or inpatient psychiatric care does not invalidate our results.

Understanding the relationship between ethnicity and mental service use is not straightforward due to the ways in which ethnicity is recorded and categorised in healthcare records of the UK. The Government Statistical Service 2022 guidelines recommend using five harmonised categories and avoiding binary categories (‘white’ and ‘other than white’) which are not analytically useful.^13^ However, this does not solve the problems of ethnicity coding in UK electronic health records, including WSIC/Discover Now, which are mainly the following: (a) registration of ethnicity is optional and can only occur when using health services, so healthier people tend to have more missing values for ethnicity than less healthy people and (b) patients may have their ethnicity recorded more than once and with discordant codes throughout their medical history, in which case ethnicity is often referred to as ‘mixed’ by data managers. Thus, less healthy people, who tend to use health services more often, tend to have more codes for ethnicity, which increases the possibility of codes being discordant, and which increases the possibility of ethnicity being recorded as ‘mixed’. This happens to a lesser extent to healthier people, who tend to use health services less. Our analysis confirms those two distortions: we found the lowest utilisation rates in people with missing data for ethnicity, and the highest rates in people with mixed ethnicity.

### Conclusion

In conclusion, we used electronic anonymised healthcare data to estimate the mental healthcare needs of CYP from deprived areas of Northwest London and found that significant need may be unmet, in line with previous data. More information is needed to understand these differences in referral rates at a granular level and the extent to which these are determined by patient level factors or system factors including service provision and referral practices. Data such as these can be used to inform strategic priorities at a population level and identify populations at particular risk. The present study addresses a key priority for the Northwest London Integrated Care Board and can directly inform local policies.

## Data Availability

Data are available in a public, open access repository.
