# Supplementary material for: Inequalities in mental health service utilisation by children and young people: a population survey using linked electronic health records from Northwest London, UK
Source: J Epidemiol Community Health. 2023 Dec 12;78(3):191–8. doi: 10.1136/jech-2023-221223 (PMC11045361; doi:10.1136/jech-2023-221223)
Supplement: Supplementary data [file jech-2023-221223supp001.pdf]

Lazzarino AI et al. Inequalities in mental health service utilisation by children and young people: a population survey using linked electronic health records from Northwest London, United Kingdom. Journal of Epidemiology ad Community Health. 2023

Appendix 1

Output from a multiple logistic regression model showing mutually-adjusted odds ratios for the utilisation of outpatient mental health care services in 2021 (OR), with 95% confidence intervals and P values, reflecting a sensitivity analysis on Table 3. As opposed to the main analysis shown in Table 3, an interaction parameter between gender and age treated as a categorical variable was included.

Factor	Category	OR	(95%CI)	P
Borough	Ealing	1	(reference category)	
	Brent	0.80	(0.71 –0.90)	<0.001
	Hillingdon	0.87	(0.77 –0.99)	0.035
	Hounslow	1.95	(1.73 –2.20)	<0.001
	Harrow	1.14	(0.97 –1.34)	0.123
	Hammersmith & Fulham	1.29	(1.13 –1.46)	<0.001
	Westminster	1.74	(1.52 –1.99)	<0.001
	Kensington & Chelsea	1.58	(1.37 –1.83)	<0.001
IMD (1=most deprived; 5=least deprived)	1 quintile increase	0.95	(0.92 –0.97)	<0.001
Interaction Borough X IMD (Slope of IMD for each Borough)	Ealing	1	(reference category)	
	Brent	0.97	(0.93 –1.02)	0.191
	Hillingdon	0.99	(0.95 –1.03)	0.475
	Hounslow	0.88	(0.85 –0.92)	<0.001
	Harrow	0.97	(0.93 –1.02)	0.242
	Hammersmith & Fulham	0.86	(0.83 –0.90)	<0.001
	Westminster	0.79	(0.75 –0.82)	<0.001
	Kensington & Chelsea	0.77	(0.73 –0.81)	<0.001
Ethnicity	White	1	(reference category)	
	Asian	0.44	(0.42 –0.45)	<0.001
	Other	0.62	(0.59 –0.65)	<0.001
	Black	0.80	(0.76 –0.85)	<0.001
	Unspecified	0.15	(0.13 –0.17)	<0.001
	Mixed	1.24	(1.17 –1.31)	<0.001
Gender & Age	Male 5–12	1	(reference category)	
	Female 5–12	0.57	(0.53 –0.60)	<0.001
	Female 13–17	3.23	(3.07 –3.40)	<0.001
	Female 18–24	1.33	(1.26 –1.40)	<0.001
	Male 13–17	1.83	(1.73 –1.93)	<0.001
	Male 18–24	0.92	(0.87 –0.98)	0.008

Lazzarino AI, *et al. J Epidemiol Community Health* 2024; 78:191–198. doi: 10.1136/jech-2023-221223
